# Serum S100A12 in the clinical diagnosis of sepsis-induced myocardial dysfunction: an integrated bioinformatics and clinical data analysis

**DOI:** 10.3389/fcvm.2025.1640788

**Published:** 2025-08-25

**Authors:** Fang Wu, Helin Hong, Ye Tian, Xiaoyan Wang

**Affiliations:** Department of Emergency, The First People’s Hospital of Guiyang, Guiyang, China

**Keywords:** sepsis, myocardial dysfunction, S100A12, inflammatory biomarker, receiver operating characteristic curve, logistic regression, bioinformatics analysis

## Abstract

**Objective:**

Sepsis is a common and life-threatening syndrome in intensive care units, frequently accompanied by myocardial dysfunction, which significantly worsens patient outcomes. S100A12, a calcium-binding protein associated with inflammation, is upregulated in various inflammatory conditions. However, its role in sepsis and related cardiac injury remains unclear.

**Methods:**

This study performed differential expression analysis using datasets from GEO to evaluate changes in S100A12 expression in sepsis and sepsis-induced myocardial dysfunction (SIMD), followed by GO and KEGG pathway enrichment analyses. Patients diagnosed with sepsis were assigned into SIMD and non-SIMD groups, along with healthy controls. Serum S100A12 expression was evaluated by ELISA and RT-qPCR. Correlations with cardiac enzymes, inflammatory markers, and cardiac function indicators were assessed.

**Results:**

Bioinformatics analysis showed upregulation of S100A12 in sepsis and SIMD, enriched in multiple inflammation-related pathways. Clinically, S100A12 mRNA and protein levels were higher in the SIMD group. There was a positive association between S100A12 concentrations and cTnI, CK-MB, PCT, and IL-6, whereas MAP and LVEF exhibited a negative correlation. Logistic regression identified S100A12 as an independent risk factor for SIMD.

**Conclusion:**

As an inflammatory biomarker, S100A12 has independent predictive value, and its combination with cardiac enzymes enables the development of an efficient clinical warning model. The study highlights a potential new biomarker and treatment focus that could aid in early detection and management of sepsis-related cardiac injury.

## 1 Introduction

Sepsis is a systemic inflammatory syndrome triggered by infection, characterized by rapid onset, fast progression, and high mortality. It remains one of the leading causes of death in intensive care units worldwide (1). Approximately 30%–60% of sepsis patients develop varying degrees of sepsis-induced myocardial dysfunction (SIMD) during the disease course. This complication severely impairs organ perfusion and worsens clinical outcomes, serving as an independent risk factor for sepsis-related mortality (2). Currently, early identification of SIMD relies primarily on echocardiography (3), sometimes in combination with conventional cardiac biomarkers such as cardiac troponin I (cTnI) (4, 5), and creatine kinase-MB (CK-MB) (6). However, these methods lack sufficient sensitivity and specificity and often fail to reflect pathological changes in real time, highlighting the urgent need for novel molecular biomarkers to improve diagnostic and prognostic capabilities.

Scientific interest has recently shifted toward understanding the impact of inflammation-related markers in organ damage caused by sepsis (7, 8). Notably, therapeutic inhibition of S100A8/A9 has been shown to attenuate both myocardial and systemic inflammation, thereby alleviating sepsis-induced cardiac dysfunction (9). Interestingly, S100A12, another key member of the S100 calcium-binding protein family, is primarily secreted by activated neutrophils and monocytes. It is markedly upregulated in various pathological states including infection (10), inflammation, and endothelial dysfunction (11–13). Through activation of the receptor for advanced glycation end-products (RAGE), S100A12 stimulates nuclear factor-κB (NF-κB) signaling and the release of multiple pro-inflammatory cytokines, thereby exacerbating systemic inflammation (14, 15). Existing studies have linked S100A12 levels to mortality of sepsis (16) and organ dysfunction in sepsis (17, 18). However, its specific involvement in the pathogenesis and clinical diagnosis of SIMD remains largely unexplored.

With the advent of high-throughput sequencing and the availability of public databases, bioinformatics tools such as those provided by Gene Expression Omnibus (GEO) offer valuable opportunities to identify novel molecular mechanisms and targets related to sepsis and its complications. Nevertheless, studies integrating bioinformatics screening with clinical validation remain scarce, which limits the translational potential of candidate biomarkers.

Therefore, this study aims to investigate the diagnostic value of S100A12 in sepsis-induced myocardial dysfunction. We identified upregulation of S100A12 in SIMD through GEO database analysis and further validated these findings in clinical serum samples. By employing RT-qPCR combined with enzyme-linked immunosorbent assay (ELISA), we quantified S100A12 mRNA and protein levels, assessed their correlations with cardiac enzymes, inflammatory cytokines, and echocardiographic parameters, and explored their diagnostic potential via logistic regression and receiver operating characteristic (ROC) curve modeling. This study not only reveals a possible mechanistic link between S100A12 and septic cardiac injury but also supports its utility as an early diagnostic biomarker, potentially offering a new strategy for timely management of SIMD. Although S100A12 has been extensively investigated in other cardiac conditions such as heart failure, Kawasaki disease, and myocardial infarction, studies specifically addressing its role in sepsis-induced myocardial dysfunction (SIMD) are scarce. This gap highlights the novelty and clinical relevance of our investigation.

## 2 Materials and methods

### 2.1 Dataset acquisition and bioinformatics analysis

Based on publicly available datasets from the Gene Expression Omnibus (GEO) database, specifically GSE65682 and GSE79962, differentially expressed genes (DEGs) associated with sepsis and SIMD were identified. Differential gene expression analysis was performed using the “limma” package in R software. DEGs meeting the cutoff criteria (absolute log₂ fold change >2 and adjusted *p*-value <0.05) were analyzed using GO term and KEGG pathway enrichment to investigate their functional characteristics and pathway involvement. These analyses were conducted using the “clusterProfiler” package in R, with a significance threshold of adjusted *p*-value < 0.05.

### 2.2 Study population and grouping

From March 2020 to December 2023, two hundred seventeen sepsis patients treated in the intensive care unit of the First People's Hospital of Guiyang participated in the study. Patients were classified into two groups based on the presence of myocardial dysfunction: the SIMD group (*n* = 106) and the non-SIMD group (*n* = 111). Sixty healthy volunteers completing routine medical examinations during the parallel period were selected as the control. Sepsis was diagnosed according to the *Sepsis-3* criteria (19). Diagnosis of myocardial dysfunction was based on clinical symptoms, transthoracic echocardiography, and laboratory findings, including reduced left ventricular ejection fraction (LVEF) and elevated myocardial enzymes. Exclusion criteria included: history of severe cardiovascular disease, malignancy, autoimmune disorders, or recent major surgery. The study has been approved by the ethics committee of the First People's Hospital of Guiyang (No. 2025-S082), and the signed informed consent forms have been obtained from the direct relatives of all the study subjects involved. SIMD was defined according to established diagnostic criteria, combining echocardiographic findings—such as reduced left ventricular ejection fraction (LVEF) and abnormal wall motion—with elevated cardiac biomarkers including cTnI and CK-MB, in the absence of alternative causes for myocardial depression. All transthoracic echocardiographic examinations were performed within 24 h of ICU admission by two senior cardiologists who were blinded to patient group allocation. A standardized imaging protocol was applied to minimize inter-operator variability, and discrepant results were resolved by consensus review.

### 2.3 Sample size estimation

Prior to the study, a power analysis was performed based on preliminary experimental data showing differences in S100A12 protein levels between the SIMD and non-SIMD groups. Using PASS software or G*Power 3.1, and assuming a two-sided test with *α* = 0.05 and power (1-*β*) = 0.90, the minimum sample size per group was calculated. Based on pretest means (SIMD: 19.6 ± 3.4 ng/ml; non-SIMD: 14.8 ± 2.9 ng/ml), at least 52 subjects were needed per group. Considering potential bias, loss to follow-up, and the demand for statistical analyses, more than 100 subjects were ultimately included in each group.

### 2.4 Sample collection and detection

Peripheral venous blood (5 ml) was collected from each subject within 24 h of ICU admission. Serum was isolated by centrifugation and stored at −80°C until analysis.

For quantitative Real-Time PCR (qRT-PCR), total RNA was extracted from serum samples, and complementary DNA (cDNA) was synthesized via reverse transcription. qRT-PCR was performed using the SYBR Green method on an ABI 7500 Real-Time PCR System. As an internal control, glyceraldehyde-3-phosphate dehydrogenase (GAPDH) was utilized, and gene expression was analyzed using the comparative Ct method.

For enzyme-Linked Immunosorbent Assay (ELISA), S100A12 protein levels in serum were measured using a commercial S100A12 ELISA kit (abcam, Lot No.ab213822). Optical density was read at 450 nm, and protein concentrations (ng/ml) were calculated accordingly.

Within detection of other clinical indicators, standardized procedures were used to assess cTnI, CK-MB, procalcitonin (PCT), C-reactive protein (CRP), interleukin-6 (IL-6), lactate, mean arterial pressure (MAP), LVEF, and Sequential Organ Failure Assessment (SOFA) score.

### 2.5 Correlation and regression analyses

Pearson correlation coefficients were calculated to assess the relationships between S100A12 expression and clinical indicators, including myocardial enzymes (cTnI, CK-MB), inflammatory markers (PCT, CRP, IL-6), and cardiac function parameters (MAP, LVEF).

Subsequently, to determine independent risk factors for SIMD, we performed multivariate logistic regression modeling. Variables included in the model were S100A12 mRNA and protein levels, myocardial enzymes, lactate, and SOFA score. Variables were retained if *p* < 0.1 and excluded if *p* > 0.05. Stepwise regression was used for model construction.

### 2.6 Model evaluation

Receiver Operating Characteristic (ROC) curves were plotted to assess the diagnostic performance of individual and combined models. Diagnostic efficacy was evaluated by calculating the area under the curve (AUC), sensitivity, and specificity. The model's calibration was evaluated through the Hosmer-Lemeshow (HL) goodness-of-fit test, with *p* > 0.05 indicating good calibration. Decision Curve Analysis (DCA) was further performed to evaluate the net clinical benefit across different threshold probabilities and compare the clinical utility of different models.

### 2.7 Data analysis

Statistical processing was conducted with SPSS 26.0, GraphPad Prism 9.0, and R version 4.2.2. Continuous measures followed either parametric (mean ± standard deviation, independent *t*-test) or non-parametric [median (IQR), Mann–Whitney test] approaches based on distributional assumptions. Count data were presented as *n* (%) and analyzed via chi-square tests. For multiple group comparisons, one-way analysis of variance (ANOVA) or Kruskal–Wallis *H* test was used, with Bonferroni correction for *post hoc* analyses. A two-sided *p*-value < 0.05 was considered statistically significant.

## 3 Results

### 3.1 Bioinformatics analysis reveals significant upregulation of S100A12 in sepsis and myocardial dysfunction

**Figure 1:**
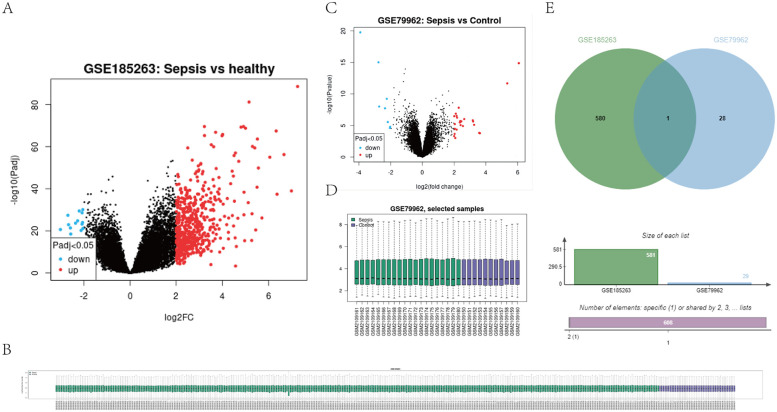
Expression profile of S100A12 in sepsis and SIMD based on GEO datasets (e.g., GSE65682, GSE79962). **(A)** Shows a volcano plot for GSE185263, indicating gene expression differences between sepsis and healthy samples. **(B)** Displays a sample clustering heatmap for GSE185263. **(C)** Presents a volcano plot for GSE79962, comparing sepsis and control samples. **(D)** Shows a box plot of selected samples from GSE79962. **(E)** Features a Venn diagram comparing datasets GSE185263 and GSE79962, indicating one shared element and specific numbers of unique elements in each set. (|log2FC| > 2, adjusted *P* < 0.05). Differential expression analysis was performed using the “limma” package in R.

To explore the potential role of S100A12 in sepsis and SIMD, differential expression analysis was conducted using GSE65682 and GSE79962 databases. The results showed that S100A12 expression was significantly upregulated in patients with sepsis and SIMD (|log₂ FC| > 2, adjusted *p* < 0.05) (Figure 1).

Further GO and KEGG enrichment analyses displayed that upregulated genes coordinately expressed with S100A12 were particularly abundant in biological processes related to inflammation, including positive regulation of MAPK activity, NF-κB signaling pathway, inflammatory response regulation, and RAGE receptor binding (all adjusted *p* < 0.05) (Table 1). These findings suggest that S100A12 may play a key role in the inflammatory mechanisms underlying SIMD.

**Table 1:** Go enrichment results of DEGs associated with S100A12 upregulation.

Ontology	ID	Description	*P-*value	*p*. adjust
BP	GO:0043406	Positive regulation of map kinase activity	0.006170213	0.020921986
BP	GO:0050729	Positive regulation of inflammatory response	0.007712766	0.020921986
BP	GO:0051092	Positive regulation of NF-kappab transcription factor activity	0.008191489	0.020921986
BP	GO:0043405	Regulation of MAP kinase activity	0.009734043	0.020921986
BP	GO:0043123	Positive regulation of I-kappaB kinase/NF-kappaB signaling	0.01	0.020921986
BP	GO:0071902	Positive regulation of protein serine/threonine kinase activity	0.01106383	0.020921986
BP	GO:0043122	Regulation of I-kappaB kinase/NF-kappaB signaling	0.013510638	0.021617021
BP	GO:0051091	Positive regulation of DNA-binding transcription factor activity	0.014095745	0.021685761
BP	GO:0007249	I-kappaB kinase/NF-kappaB signaling	0.015319149	0.021960486
BP	GO:0071900	Regulation of protein serine/threonine kinase activity	0.019787234	0.024072948
BP	GO:0050727	Regulation of inflammatory response	0.020957447	0.024072948
BP	GO:0045860	Positive regulation of protein kinase activity	0.02106383	0.024072948
BP	GO:0051090	Regulation of DNA-binding transcription factor activity	0.024042553	0.025307951
BP	GO:0033674	Positive regulation of kinase activity	0.025319149	0.025968358
BP	GO:0043410	Positive regulation of MAPK cascade	0.026117021	0.026117021
MF	GO:0050786	RAGE receptor binding	0.000543183	0.001629549

Significantly enriched BP and MF are listed. Multiple testing was corrected using the Benjamini-Hochberg method, with a significance threshold set at adjusted *P*-value < 0.05.

### 3.2 S100A12 levels are elevated in the serum of SIMD patients

Two hundred seventeen confirmed sepsis cases were included in our analysis, and classified into SIMD group (*n* = 106) and the non-SIMD group (*n* = 111). In addition, 60 healthy volunteers served as the control group. No significant variations were found between the groups regarding demographic characteristics including gender, age, BMI, or comorbidities (e.g., hypertension, diabetes) (all *p* > 0.05, Table 2), indicating good comparability.

**Table 2:** Baseline characteristics and S100A12 expression levels among three study groups.

Parameters		Healthy (*n* = 60)	Non-SIMD (*n* = 111)	SIMD (*n* = 106)	*X*^2^/*F*	*P* (3 groups)	*X*^2^/*t* (2 groups)	*P* (2 groups)
Age		43.47 ± 9.103	46.38 ± 10.292	47.2 ± 11.496	2.487	0.085	0.554	0.58
Sex	Male	22 (36.67%)	57 (51.35%)	47 (44.34%)	3.478	0.176	1.068	0.301
	Female	38 (63.33%)	54 (48.65%)	59 (55.66%)				
BMI		22.1 (20,23.475)	22.1 (20.7,23.7)	21.55 (20.4,22.925)	3.057	0.217	−1.799	0.072
Hypertension	Yes	21 (35.0%)	37 (33.33%)	38 (35.85%)	0.156	0.925	0.152	0.697
	No	39 (65.0%)	74 (66.67%)	68 (64.15%)				
	Yes	11 (18.33%)	15 (13.51%)	11 (10.38%)	2.099	0.350	0.506	0.477
	No	49 (81.67%)	96 (86.49%)	95 (89.62%)				
S100A12 mRNA		1.03 ± 0.31	1.79 ± 0.41	2.58 ± 0.48	272.35	0	13.113	0
S100A12 Protein		10.33 ± 1.97	14.79 ± 2.92	19.60 ± 3.36	200.815	0	11.293	0

**Figure 2:**
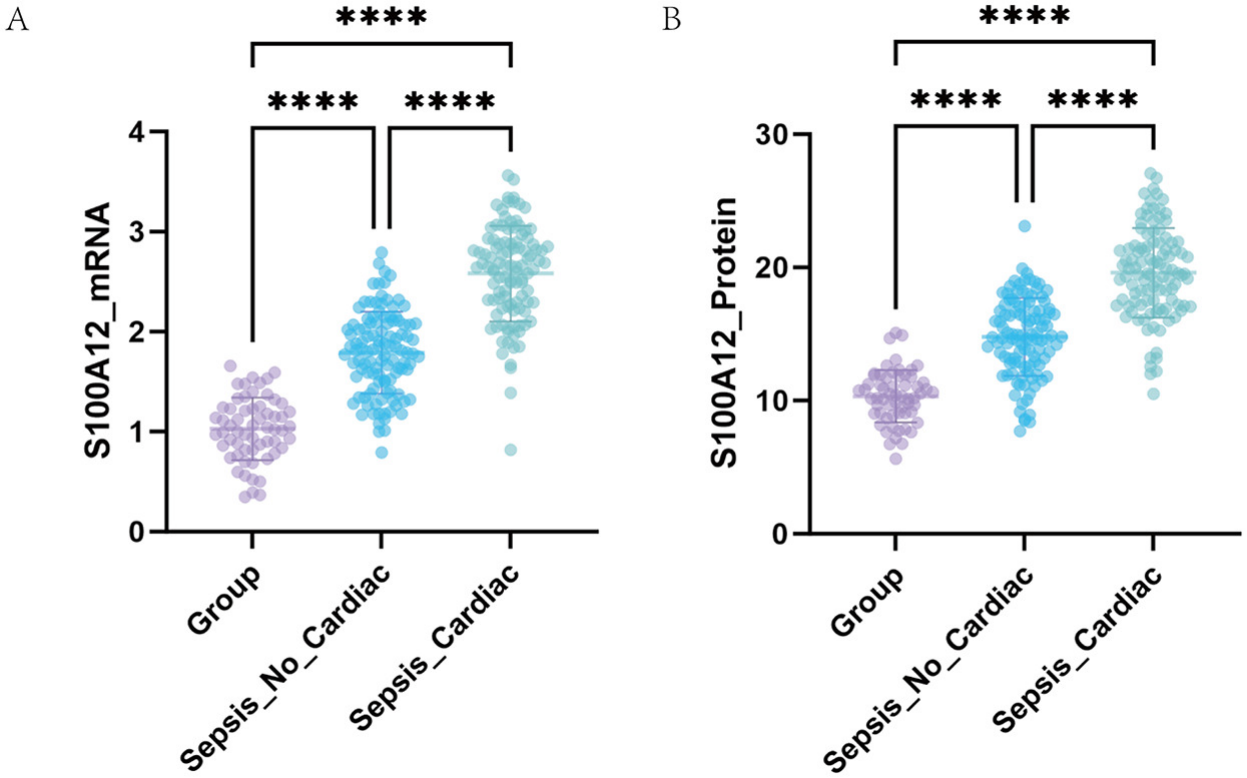
Expression of S100A12 detected by qRT-PCR and ELISA. (A) Comparison of serum S100A12 mRNA levels among different groups. (B) Comparison of serum S100A12 protein levels among different groups.

At the molecular level, serum S100A12 mRNA expression was higher in the SIMD group (2.58 ± 0.48) than in the non-SIMD group (1.79 ± 0.41) and the healthy control group (1.03 ± 0.31) (all *p* < 0.001) (Figure 2A). Additionally, the non-SIMD group exhibited higher S100A12 expression (*p* < 0.001).

At protein level, serum S100A12 protein concentration was highest in the SIMD group (19.60 ± 3.36 ng/ml), followed by the non-SIMD group (14.79 ± 2.92 ng/ml), and lowest in the control group (10.33 ± 1.97 ng/ml), with highly significant differences between groups (all *p* < 0.001) (Figure 2B). These findings indicate a stepwise increase in S100A12 expression in sepsis patients, closely associated with the presence of myocardial dysfunction.

### 3.3 S100A12 expression correlates with cardiac function-related parameters

**Figure 3:**
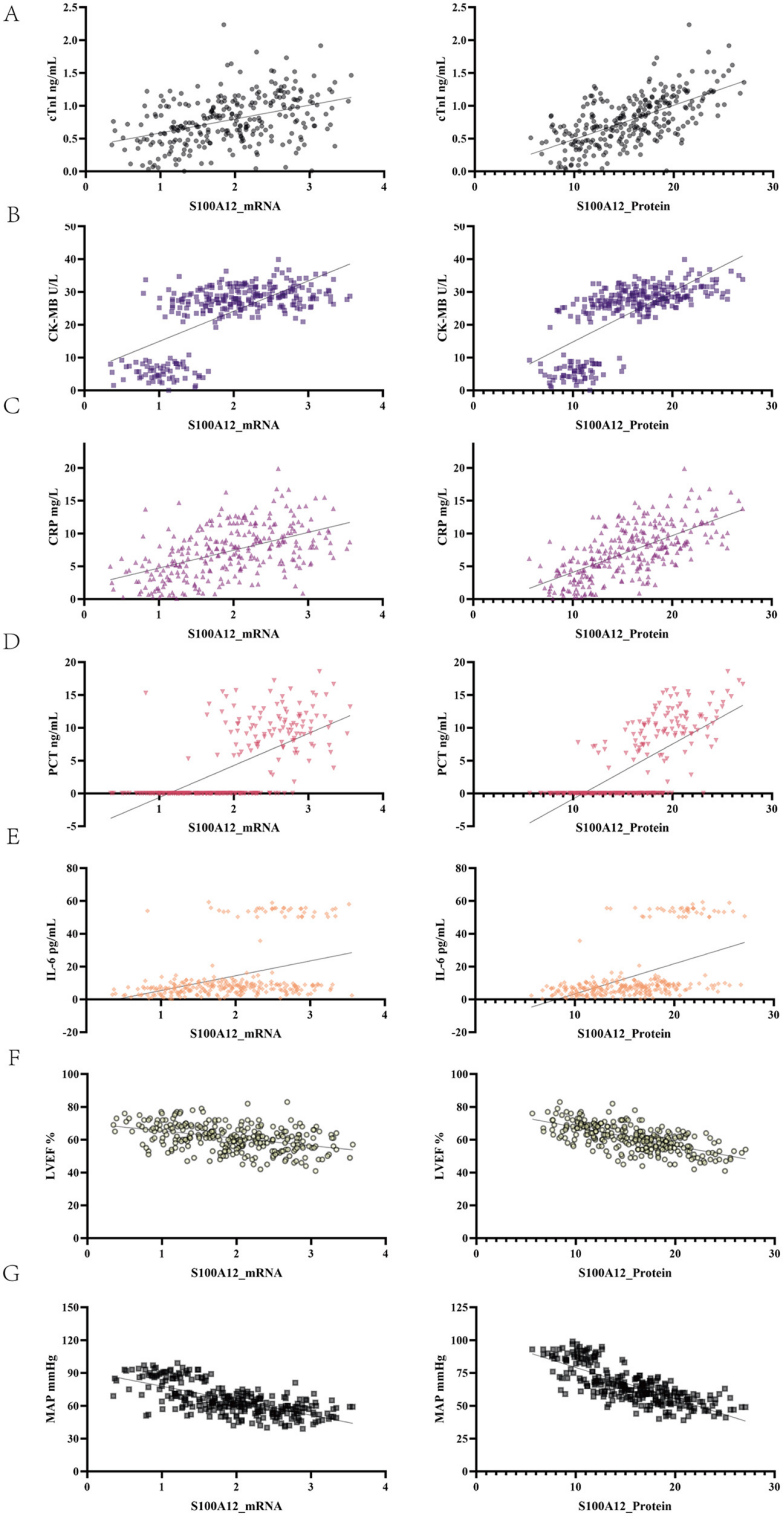
Correlation between S100A12 expression and indicators of myocardial injury, inflammation, and cardiac function. The left column shows scatter plots of Pearson correlations between S100A12 mRNA and each indicator, while the right column shows those for S100A12 protein expression. **(A)** cTnI. **(B)** CK-MB. **(C)** CRP. **(D)** PCT. **(E)** IL-6. **(F)** LVEF. **(G)** MAP.

To further investigate the clinical significance of S100A12 in SIMD, Pearson correlation analysis was performed between S100A12 levels and myocardial enzymes, inflammatory markers, and cardiac function parameters (Table 3; Figure 3). S100A12 mRNA expression showed a moderate positive correlation with cTnI and CK-MB levels (*r* = 0.4065 and 0.6696, respectively; both *p* < 0.001). S100A12 protein levels were strongly positively correlated with these myocardial enzymes, particularly CK-MB (*r* = 0.7069, *p* < 0.001). Regarding inflammation, both mRNA and protein levels of S100A12 showed significant positive correlations with CRP, PCT, and IL-6, with the strongest correlation observed between S100A12 protein and PCT (*r* = 0.7182). Additionally, S100A12 expression was negatively correlated with cardiac function indicators. Specifically, S100A12 mRNA was negatively correlated with LVEF and MAP (*r* = −0.408 and −0.6762, respectively; both *p* < 0.001), while the protein expression of S100A12 showed a strong negative correlation with MAP (*r* = −0.7735, *p* < 0.001). These results suggest that S100A12 may be involved in the pathogenesis of SIMD.

**Table 3:** Correlation analysis between S100A12 expression and clinical indicators of myocardial injury, inflammation, and cardiac function.

Parameters	S100A12 mRNA		S100A12 protein	
	*r*	*p*	*r*	*p*
cTnI (ng/ml)	0.4065	<0.0001	0.6322	<0.0001
CK_MB (U/L)	0.6696	<0.0001	0.7069	<0.0001
CRP (mg/L)	0.5067	<0.0001	0.6604	<0.0001
PCT (ng/ml)	0.6602	<0.0001	0.7182	<0.0001
IL-6 (pg/ml)	0.385	<0.0001	0.4971	<0.0001
LVEF (%)	−0.408	<0.0001	−0.6479	<0.0001
MAP (mmHg)	−0.6762	<0.0001	−0.7735	<0.0001

Pearson correlation analysis was performed to report the correlation coefficients (*r*) and corresponding *P*-values for S100A12 mRNA and protein expression. A *p*-value < 0.05 was considered statistically significant.

### 3.4 S100A12 is an independent risk factor for SIMD

To further clarify the clinical role of S100A12, a comparative analysis of serum lactate concentrations and SOFA scores was performed between the two sepsis cohorts. The SIMD group had higher lactate levels (3.09 ± 1.23 vs. 2.70 ± 1.16, *p* = 0.016) and SOFA scores (median 8 vs. 7, *p* = 0.002) (Table 4).

**Table 4:** Comparison of lactate levels and SOFA scores between two patient groups.

Index	Non-SIMD (*n* = 111)	SIMD (*n* = 106)	Z/*t*	*P*
Lactate level	2.6997 ± 1.16491	3.0927 ± 1.22679	2.421	0.016
SOFA score	7 (5, 9)	8 (6, 9)	3.063	0.002

**Figure 4:**
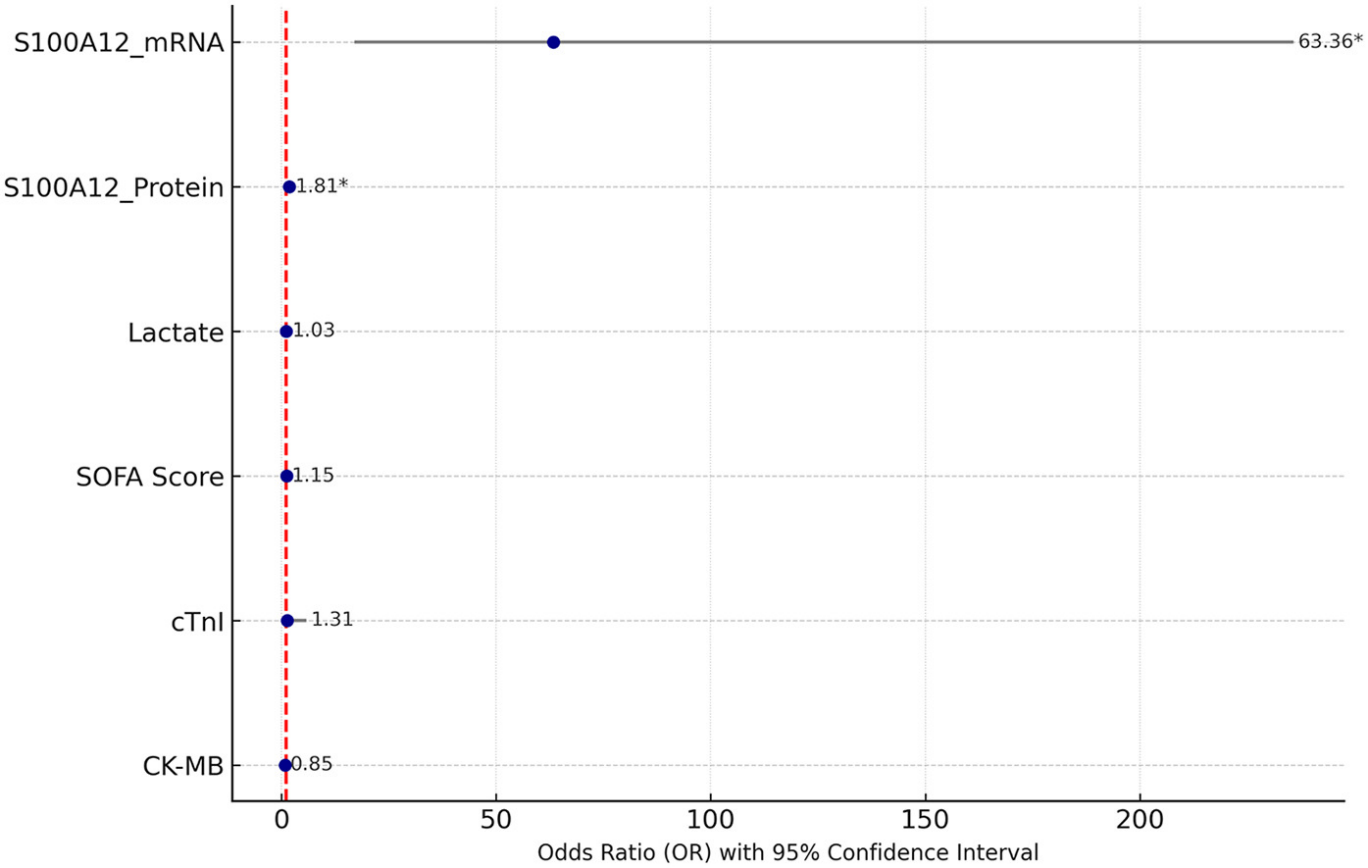
Forest plot of multivariate logistic regression analysis. The figure shows the OR values of each variable and their 95% *CI*, with significant variables marked by an asterisk (*).

Multivariate logistic regression analysis was performed including S100A12 mRNA and protein levels, lactate, SOFA score, cTnI, and CK-MB. As shown in Table 5; Figure 4, S100A12 remained an independent risk factor for SIMD. Specifically, the *OR* for S100A12 mRNA was 63.364 (95% *CI*: 17.026–235.812, *p* < 0.001), and for S100A12 protein it was 1.808 (95% *CI*: 1.432–2.283, *p* < 0.001). In contrast, lactate, SOFA score, cTnI, and CK-MB did not reach statistical significance in the multivariate model (*p* > 0.05 for all). These findings suggest that S100A12 has independent predictive value for early risk identification of SIMD.

**Table 5:** Multivariate logistic regression analysis of S100A12 as an independent predictor in SIMD.

Variable	*β*	SE	Wald	*p*-value	*OR*	95% *CI* (lower limit, superior limit)
S100A12_mRNA	4.149	0.67	38.289	0	63.364	17.026–235.812
S100A12_Protein	0.592	0.119	24.766	0	1.808	1.432-2.283
Lactate level	0.029	0.2	0.022	0.883	1.03	0.695–1.525
SOFA score	0.142	0.108	1.739	0.187	1.152	0.933–1.423
cTnI	0.27	0.763	0.125	0.723	1.31	0.294–5.845
CK_MB	−0.163	0.09	3.248	0.072	0.85	0.711–1.014
Constant	−15.9	2.809	32.034	0	0	

Included variables: S100A12 mRNA, protein expression, lactate, SOFA score, cTnI, CK-MB. *P* < 0.05 indicates statistical significance.

### 3.5 Diagnostic performance of S100A12-based predictive models for SIMD

**Figure 5:**
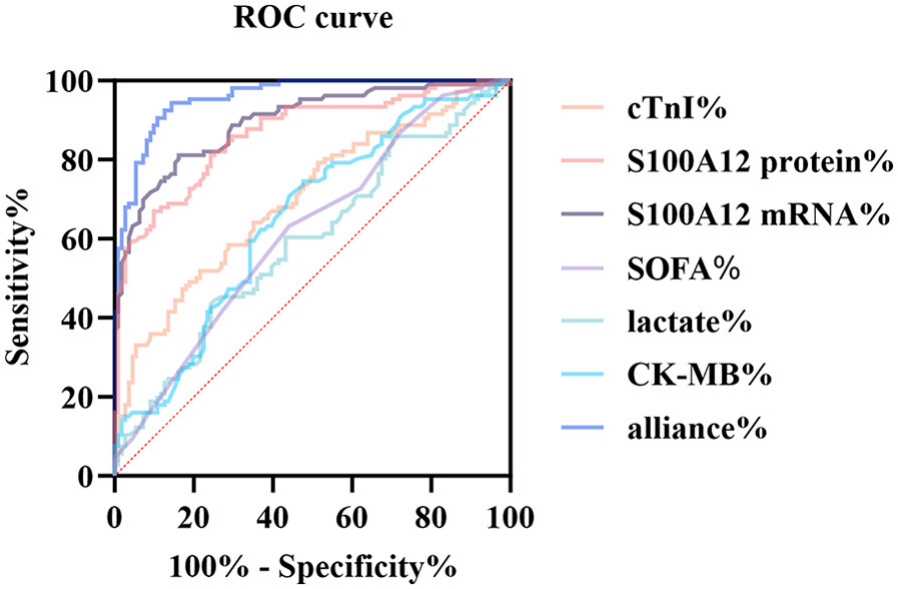
ROC curve comparison of multiple biomarkers for diagnosing SIMD. The figure displays the ROC curves of S100A12 mRNA, S100A12 protein, cTnI, CK-MB, lactate, SOFA score, and the combined prediction model (alliance) for identifying sepsis-induced myocardial dysfunction.

To assess the diagnostic value of S100A12 for sepsis-associated myocardial dysfunction, we performed ROC curve evaluation. ROC curve analysis indicated excellent discriminative capacity for S100A12 mRNA (AUC 0.895, 95% *CI* 0.853–0.937; sensitivity 81.1%, specificity 83.8%). The protein counterpart maintained good diagnostic value (AUC 0.865, 95% *CI* 0.817–0.914; sensitivity 82.1%, specificity 74.8%). In comparison, the AUCs for cTnI and CK-MB were 0.6997 and 0.6491, respectively, with significantly lower diagnostic accuracy. Lactate and SOFA scores also demonstrated limited diagnostic value. The standalone diagnostic performance of S100A12 mRNA and protein is superior to that of traditional markers cTnI and CK-MB (Table 6; Figure 5).

**Table 6:** ROC analysis results of S100A12 and conventional indicators in diagnosing septic myocardial dysfunction.

Variable	AUC	Youden	S.E.	95% *CI*	Sensitivity (%)	Specificity (%)
S100A12 mRNA	0.8952	0.6491	0.02133	0.8534–0.9370	81.13	83.78
S100A12 protein	0.8652	0.5685	0.02466	0.8168–0.9135	82.08	74.77
cTnI	0.6997	0.2901	0.03531	0.6305–0.7689	64.15	64.86
CK-MB	0.6491	0.2678	0.03720	0.5762–0.7220	74.53	52.25
Lactate level	0.5917	0.1714	0.03855	0.5162–0.6673	60.38	56.76
SOFA score	0.6193	0.1907	0.03778	0.5452–0.6933	63.21	55.86
Constant (Combined)	0.9576	0.7902	0.01182	0.9344–0.9808	94.34	84.68

Metrics such as AUC, standard error, Youden index, sensitivity, and specificity were included. The combined prediction model is a logistic model constructed based on S100A12, cTnI, and CK-MB. The closeness of AUC to 1 indicates better predictive performance.

### 3.6 Combined model incorporating S100A12 and cardiac function indicators demonstrates high clinical applicability

**Figure 6:**
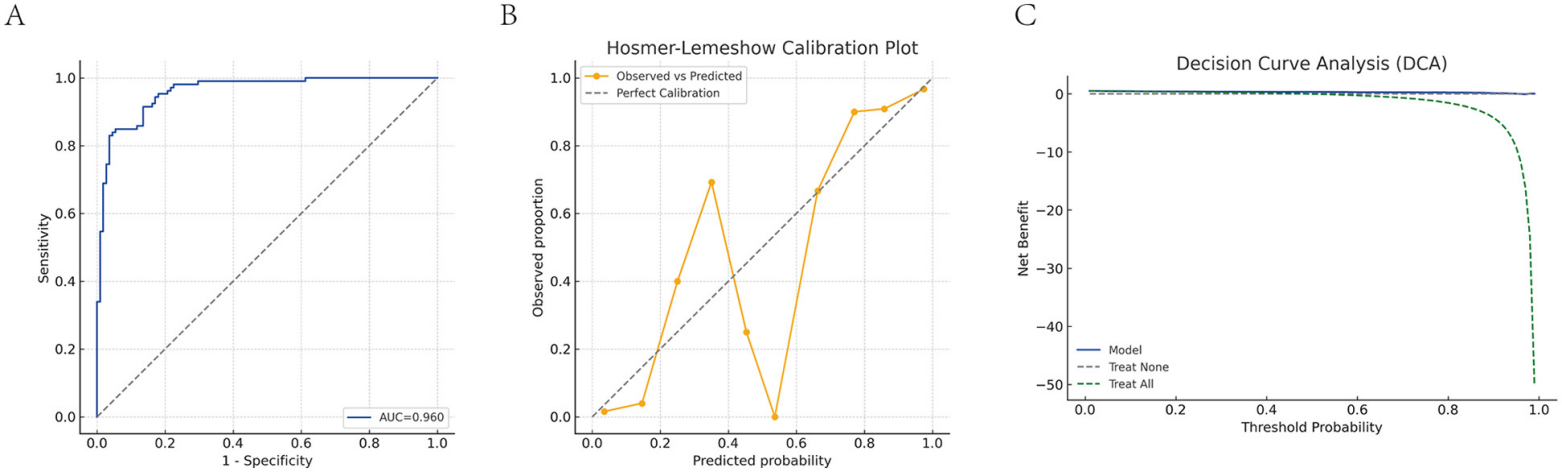
Diagnostic performance evaluation of the combined predictive model. **(A)** ROC curve analysis of the combined model. **(B)** HL calibration plot. **(C)** DCA of the combined model. The blue solid line represents the net benefit of the combined prediction model (S100A12 mRNA + S100A12 protein + LVEF + MAP) across different threshold probabilities; the green dashed line represents the “treat all” strategy (intervene on all patients); the gray dashed line represents the “treat none” strategy (no intervention for anyone). When the model curve is above these two dashed lines, it indicates a positive clinical net benefit.

A final logistic regression model was developed incorporating S100A12 mRNA, protein levels, and cardiac function indicators (LVEF, MAP). This model demonstrated excellent discrimination ability, with an AUC of 0.960 (Figure 6A). Model calibration was assessed using the HL test, yielding an HL statistic of 7.39 and *P* = 0.495 (Figure 6B), indicating good agreement between predicted and observed values. DCA was utilized to determine the threshold-dependent clinical value of the predictive model. As shown in Figure 6C, the blue curve representing the model was consistently above the “treat all” and “treat none” curves across most probability thresholds, demonstrating its strong clinical benefit and potential to support decision-making in practice.

## 4 Discussion

SIMD is one of the most common and challenging complications in critical care, yet sensitive and reliable molecular markers for early identification remain lacking (20). This study combined bioinformatics analysis with clinical validation and found that S100A12 was persistently elevated in patients with sepsis and SIMD. Elevated S100A12 was significantly correlated with myocardial enzymes, inflammatory markers, and hemodynamic parameters, demonstrating independent predictive value. A diagnostic model incorporating S100A12 achieved an AUC of 0.9576, outperforming traditional indicators, suggesting strong potential for use in early clinical warning systems.

Compared with previous studies, this research is the first to systematically validate S100A12 within a clinical diagnostic framework for SIMD. Previous literature has identified S100A12 as a pro-inflammatory damage-associated molecular pattern, capable of binding to receptors such as RAGE (receptor for advanced glycation end-products) and TLR4, leading to downstream activation of the NF-κB and MAPK signaling pathways. These cascades result in increased transcription of pro-inflammatory cytokines (e.g., TNF-α, IL-6, IL-1β), endothelial dysfunction, and leukocyte recruitment, which together exacerbate tissue inflammation and injury (10, 11, 13, 21, 22). Our data not only reaffirmed these associations but also demonstrated that S100A12 levels correlated positively with cardiac injury markers (cTnI, CK-MB) and inflammatory mediators (IL-6, PCT), while showing negative correlation with LVEF and MAP, key indicators of cardiac function and hemodynamic stability. The GO and KEGG enrichment analyses further indicated significant involvement of S100A12-related genes in immune-inflammatory signaling, particularly MAPK, NF-κB, and Toll-like receptor pathways, all of which are known to be central in SIMD pathogenesis. Specifically, activation of NF-κB signaling has been shown to drive cardiomyocyte apoptosis and mitochondrial dysfunction under septic conditions, contributing to contractile impairment (23). MAPK signaling, especially through p38 and JNK sub-pathways, is implicated in the regulation of myocardial inflammation, calcium overload, and reactive oxygen species accumulation during systemic inflammatory responses (24).

S100A12, through its RAGE interaction, may serve as an upstream amplifier of these pro-death signals, forming a feed-forward loop that sustains and intensifies myocardial injury. Moreover, recent studies have highlighted that S100A12 can modulate endothelial permeability, exacerbate capillary leakage, and promote macrophage activation, all of which further contribute to the microcirculatory dysfunction and myocardial oxygen supply-demand mismatch observed in SIMD. This multifaceted role aligns with our observation that elevated S100A12 is associated with reduced MAP and LVEF, suggesting a direct link between systemic inflammation, vascular collapse, and myocardial depression. Collectively, these results indicate S100A12 may serve functions beyond a diagnostic biomarker, also an active pathogenic mediator in SIMD, contributing to myocardial damage via inflammatory injury, oxidative stress, and endothelial dysfunction. The robust correlations observed in our clinical cohort provide translational relevance and support the incorporation of S100A12 into diagnostic or therapeutic frameworks for SIMD.

In terms of practical application, S100A12 has several advantages as a serum biomarker. Its expression is stable, sample collection is convenient, and it can be detected at both the mRNA level (via qRT-PCR) and the protein level (via ELISA). Compared with traditional cardiac enzymes such as cTnI and CK-MB, S100A12 demonstrated higher sensitivity and specificity in detecting SIMD, particularly in the early inflammatory phase when classical markers may still remain within normal ranges (25). This is likely because S100A12, as an alarmin of the S100/calgranulin family, is rapidly released by activated neutrophils and monocytes in response to systemic infection, serving as an early signal of innate immune activation and sterile inflammation (26). In contrast, cTnI and CK-MB primarily reflect structural myocardial injury, often representing a downstream consequence of prolonged inflammation or hypoperfusion, thus limiting their ability to detect subclinical or functional myocardial impairment. Previous studies have linked S100A12 to various cardiovascular diseases, including heart failure, ST-elevation myocardial infarction (STEMI), and coronary artery disease (CAD). Elevated S100A12 levels in these conditions have been associated with plaque instability, coronary calcification, and adverse clinical outcomes. Our findings in SIMD are consistent with these observations, suggesting that S100A12 may represent a shared inflammatory mediator across different cardiac pathologies, while also highlighting its potential as a specific biomarker for sepsis-related myocardial dysfunction.

The combined diagnostic model developed in this study incorporated S100A12 (mRNA and protein levels) together with inflammatory markers (such as IL-6 and PCT), cardiac function indicators (e.g., LVEF, MAP), and organ dysfunction scores (e.g., SOFA score). This multimodal approach significantly enhanced the discriminative power for SIMD, as evidenced by a high AUC in ROC curve analysis. From a mechanistic perspective, this combination model captures both upstream immune dysregulation (via S100A12 and IL-6), and downstream functional impairment (via LVEF and MAP), achieving temporal and pathophysiological complementarity. For instance, while S100A12 reflects early immune activation, IL-6 integrates the broader cytokine storm environment, and SOFA score quantifies multi-organ dysfunction severity. At the same time, LVEF offers a direct assessment of cardiac systolic performance, and MAP reflects hemodynamic stability and tissue perfusion. By integrating these heterogeneous but interrelated indicators, the combined model overcomes the limitations of single biomarkers and allows for more accurate identification of SIMD across its full clinical spectrum—from functional impairment without structural damage, to irreversible myocardial injury.

Such complementarity is particularly critical in the context of sepsis, where myocardial depression is often multifactorial, involving not only direct myocardial insult but also microcirculatory disturbance, cytokine-induced myocardial stunning, and oxidative stress-mediated mitochondrial dysfunction. In this framework, S100A12 acts not only as a surrogate of inflammation but also as a potential mediator of myocardial injury through RAGE-NF-κB signaling, thereby bridging the immune-inflammatory response and cardiac dysfunction. Taken together, these results suggest that S100A12-based multimarker strategies could serve as an effective early-warning system for SIMD, improving risk stratification, individualized monitoring, and potentially guiding targeted anti-inflammatory interventions in clinical practice. Decision curve analysis showed high net clinical benefit across a range of threshold probabilities, demonstrating its strong potential in clinical decision-making. This lays a foundation for future precision stratification and management of sepsis.

Despite the strengths in study design and data analysis, several limitations should be acknowledged, beginning with the retrospective nature and restricted sample size from a single institution, and further validation in multi-center, large-sample cohorts is necessary. Second, whether S100A12 acts as a pathogenic factor or is merely a reactive elevation in myocardial dysfunction remains unclear and requires further investigation using animal models and cell-based experiments. Third, this study did not include longitudinal samples, preventing an assessment of S100A12's dynamic changes over time and its relationship with patient outcomes. Future research should incorporate long-term follow-up and interventional studies to clarify its causal role and therapeutic potential. Several limitations warrant mention. First, although sample collection was standardized within 24 h of ICU admission, variability in disease onset prior to admission was not controlled and may have influenced biomarker levels. Second, while major cardiovascular diseases were excluded, data on medications such as vasopressors, corticosteroids, and the timing of antibiotic administration were not comprehensively recorded; these factors may confound biomarker interpretation and should be addressed in future prospective studies.

## 5 Conclusion

In summary, this study reveals the expression pattern of S100A12 in SIMD and its close association with inflammation and cardiac injury, confirming S100A12 as an independent predictive factor for SIMD. Beyond its superior diagnostic performance, S100A12 may also represent a potential target within the inflammation-cardiac dysfunction axis. These findings provide new insights and theoretical support for early identification and intervention in SIMD.

## Data Availability

Publicly available datasets were analyzed in this study. This data can be found here: Gene Expression Omnibus (GEO) database, accession numbers: GSE65682 and GSE79962.
